# Glutaraldehyde Cross-Linked MXene-Nanocellulose Membrane for Efficient Dye/Salt Separation

**DOI:** 10.3390/membranes15100287

**Published:** 2025-09-24

**Authors:** Yu Zhang, Ming Qiu

**Affiliations:** College of Biological Chemical Science and Engineering, Jiaxing University, Jiaxing 314001, China

**Keywords:** MXene membrane, cellulose nanocrystal, cross-linking, stability, dye/salt separation

## Abstract

This study proposes a novel approach for preparing a laminated carbides and nitrides (MXene) membrane with loose nanochannels by intercalating cellulose nanocrystal (CNC) and cross-linking with glutaraldehyde. The interlayer spacing of the MXene membrane can be expanded by one-dimensional CNC, while cross-linking with glutaraldehyde enhances the stability of the membrane. The optimized membrane displays a high water permeate flux of 96.8 L m^−2^ h^−1^ (2.1 times higher than MXene membrane) and good selectivity (methyl blue rejection rate: ~99.6%; NaCl rejection rate: <5.0%). This strategy provides a universal way to prepare high-performance two-dimensional membranes.

## 1. Introduction

The textile industry generates a large amount of wastewater containing high concentrations of dyes and salts, and direct discharge can cause serious environmental pollution [1,2,3]. Due to the fact that salt and dyes are renewable resources, it is crucial to develop effective methods for separating dye/salt mixtures from aqueous solutions [4,5]. Pressure-driven membrane separation technology, such as nanofiltration and reverse osmosis, has been widely used for dye or salt separation due to its excellent capability to separate dyes solution [6,7,8]. However, traditional thin-film composite polyamide membrane still exhibits serious problems, such as fouling and the permeate flux/selectivity trade-off [9,10]. Thus, developing novel high-performance separation membrane materials remains the primary challenge in various industrial filtration and purification processes.

In recent years, two-dimensional (2D) materials, including graphene oxide (GO), molybdenum disulfide, transition metal carbides/nitrides (MXene), metal–organic frameworks (MOFs) and covalent organic frameworks (COFs) [11,12,13,14,15] have shown promising application prospects for preparing high-performance membranes. Among them, the novel MXene known as Ti_3_C_2_T_x_ has emerged as the one of most favorable materials for effectively removing target contaminants and ions due to its unique ultra-thin structure and nanocapillary channels [12,16]. However, the strict trade-off of molecular sieves reduces the water permeate flux and the long-term structural integrity in water [17,18].

Modification is commonly used to alleviate low water flux and swelling issues [19,20]. At present, the most widely used method to improve the permeate flux of layered MXene membranes is through intercalation to adjust the interlayer d-spacing [21]. Many materials have been used as the intercalation agents, such as metal ions, organic molecules and nanomaterials [22,23,24]. Long et al. [25] prepared an Al_2_O_3_ intercalated MXene membrane with stable and tunable lamellar nanochannels. The optimized membrane exhibited an outstanding water permeate flux of 88.8 L m^−2^ h^−1^ bar^−1^ and a high dye rejection rate (>99.5% rejection rates to rhodamine B and methyl blue). Luo et al. [26] prepared an amino acid-modified MXene membrane that showed a superior anti-fouling property, with a flux recovery rate of 80.59% and high salt rejection rates. Zheng et al. [27] prepared a covalently bridged MXene/covalent organic framework (COF) hybrid membrane, which showed improved water flux (up to 300 L m^−2^ h^−1^ bar^−1^) and dye rejection rate (98.2% for Congo red). The intercalation agents would enlarge the interlayer spacing of its adjacent MXene nanosheets, thus improving the water permeate flux [28]. In addition, the intercalation agents also act as a lock through hydrogen bonding or chelation to inhibit the swelling of MXene nanosheets in water [29]. Despite the improved separation properties displayed by these MXene membranes, they possessed certain defects and insufficient mechanical strength due to the weak interaction, leading to operational instability during long-term testing [22].

Cellulose nanocrystal has been widely used for material modification due to its advantages such as low cost, high aspect ratio and biodegradability [30]. As a modifier, CNC can improve the mechanical properties of the materials [31]. In addition, the hydrophilic CNC has been proven to enhance the permeate flux and anti-fouling properties of the membrane when incorporated into the polymer membrane matrix [32,33]. All these characteristics make CNC an attractive nanocomposite membrane filler for water treatment applications.

In this study, high-performance MXene membranes were prepared by the intercalation of CNC, followed by cross-linking with glutaraldehyde. Firstly, CNC was mixed with MXene solution and then MXene-based membranes were prepared via vacuum filtration. The continuous 3D nanostructures formed between nanosheets and CNC can effectively inhibit the restacking of MXene nanosheets and adjust the interlayer spacings of the membranes. Their structure and composition were analyzed by a series of characterizations, including X-ray diffraction (XRD), water contact angle (WCA), Fourier transform infrared spectroscopy (FT-IR), zeta potential and X-ray photoelectron spectroscopy (XPS). Their morphology was observed by scanning electron microscope (SEM), transmission electron microscopy (TEM) and atomic force microscopy (AFM). Meanwhile, the composite membrane was optimized by adjusting the mass ratio between MXene and CNC. The resulting membrane shows a good combination of high permeate flux, selectivity and anti-fouling properties.

## 2. Materials and Methods

### 2.1. Materials

Polyethersulfone (PES) membranes (0.22 μm), lithium fluoride (LiF, 99%), alcian blue (AB, 50%), methyl blue (MB, AR), Congo red (CR, 98%), methylene blue (MeB, 98%), glutaraldehyde (50% in water) and Ti_3_AlC_2_ powder (400 mesh, 99.5%) were purchased from Titanchem Co., Ltd., Shanghai, China. Hydrochloric acid (HCl, 37%), sodium sulfate (Na_2_SO_4_, 99%), sodium chloride (NaCl, 99.99%), magnesium sulfate (MgSO_4_, 98%) and magnesium chloride (MgCl_2_, 99%) were purchased from Sinopharm Chemical Reagent Co., Ltd., Shanghai, China. Cellulose nanocrystal (CNC) powder (length ca. 200 nm, diameter ca. 10 nm), bovine serum albumin (BSA, 96%), dopamine hydrochloride (98%) and tris (hydroxymethyl) aminomethane hydrochloride buffer (Tris-HCl, 0.01M, pH = 8.5) were obtained from Macklin Reagent Co., Ltd., Shanghai, China. All purchased reagents were used directly.

### 2.2. Synthesis of Ti_3_C_2_T_x_ Mxene Nanosheets

The MXene nanosheet suspension is synthesized through a mild etching method [34]. Typically, 1.0 g of lithium fluoride (LiF) is dissolved in 10.0 mL of 9.0 mol/L hydrochloric acid (HCl) in a tetrafluoroethylene beaker. Then, 0.5 g of Ti_3_AlC_2_ is gradually added to the solution, and the mixture is stirred at 35 °C for 24 h. The product is centrifuged (3500 rpm for 5 min) and washed with deionized (DI) water six times until the pH value of the solution is >6. After 1 h of ultrasonic treatment (in a nitrogen environment and ice-water bath), the mixed solution is centrifuged at 4000 rpm for 1 h. The collected supernatant is stored in the refrigerator before use.

### 2.3. Fabrication of Mxene Membranes

As is standard, 0.2 g dopamine hydrochloride was added into 100.0 mL of Tris-HCl buffer (0.01 M, pH = 8.5) to prepare the polydopamine (PDA) solution. Then, the PES membranes were soaked in the polydopamine solution at room temperature for 3 h. The modification of PDA on the membrane surface can enhance the force between the substrate and MXene nanosheets.

**Figure 1:**
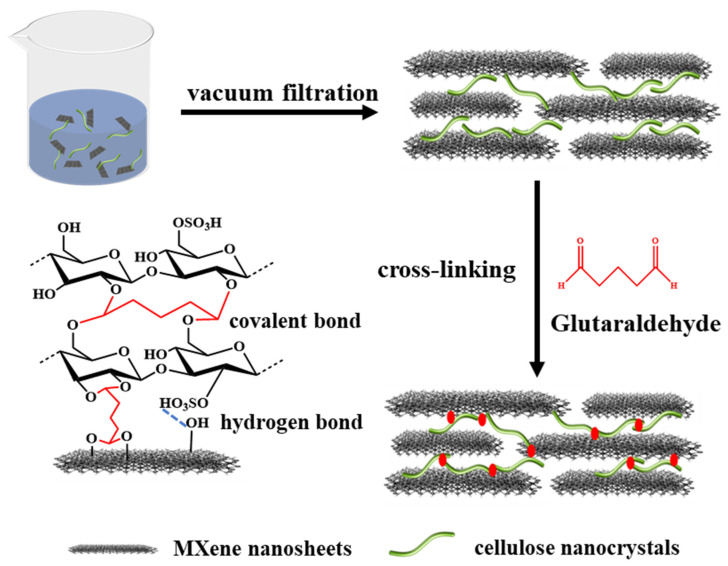
Schematic diagrams of preparation process of the membrane.

The preparation process of composite film is shown in Figure 1. Different qualities of cellulose nanocrystal were added into the 50.0 mL of MXene solutions (20.0 μg/mL), then sonicated for 0.5 h and stirred for 1 h under a nitrogen atmosphere at room temperature. The 2D membranes were prepared by depositing the mixture onto PES membranes by vacuum filtration method. Then, the composite membranes were cross-linked with 5% GA solution at 60 °C for 1 h and subsequently washed with DI water to remove unreacted GA molecules. The prepared composite membranes were named GA-CMx, where M represents the MXene, C represents CNC and x (%) represents the ratio of CNC to MXene. In this study, the values of x are 10, 20, 30 and 40, respectively. The detailed composition of the prepared membrane is shown in Table 1.

### 2.4. Characterizations

The morphology of the MXene nanosheet and the CNC were observed by transmission electron microscopy (TEM, FEI, TalosF200X, Waltham, MA, USA). The MXene membranes were characterized by X-ray diffraction (XRD, Panalytical X’Pert3 Powder, Almelo, The Netherlands) with Cu Kα radiation at a step of 1.0°. The chemical groups of the nanosheets were analyzed using Fourier transform infrared spectroscopy (FT-IR, Thermo Fisher Scientific, IS50, Waltham, MA, USA) in the range of 4000–500 cm^−1^ (scans number: 64, resolution: 1 cm^−1^, crystal type: diamond). The membranes were dried in a vacuum oven at 60 °C for 24 h prior to testing. The surface and cross-section morphology of membranes were measured uaing a scanning electron microscope (SEM, S4800, Hitachi, Tokyo, Japan). All samples were mounted on aluminum specimen stubs and sputter coated with gold before testing. Atomic force microscopy (AFM, Bruker Dimension Icon, Karlsruhe, Germany) was used to obtain a topographical image of the membrane in tapping mode. The quantitative roughness was calculated using the NanoScope Analysis V3.0 software in terms of mean roughness (R_a_). The chemical compositions of membranes were analyzed by X-ray photoelectron spectroscopy (XPS, Thermo Fisher Scientific, K-Alpha, Waltham, MA, USA). The contact angle of the membrane was studied on a contact angle measuring instrument (Dataphysics, OCA40, Stuttgart, Germany). A 5 μL drop of pure distilled water was placed on the membrane surface, using a syringe with a 22-gauge needle. The surface contact angles were the mean of five determinations. The zeta potential of the membrane was analyzed using an electrokinetic analyzer (Anton Paar, SurPASS, Graz, Austria). The experimental data were plotted with Origin 2019 software.

### 2.5. Membrane Filtration Performance

**Figure 2:**
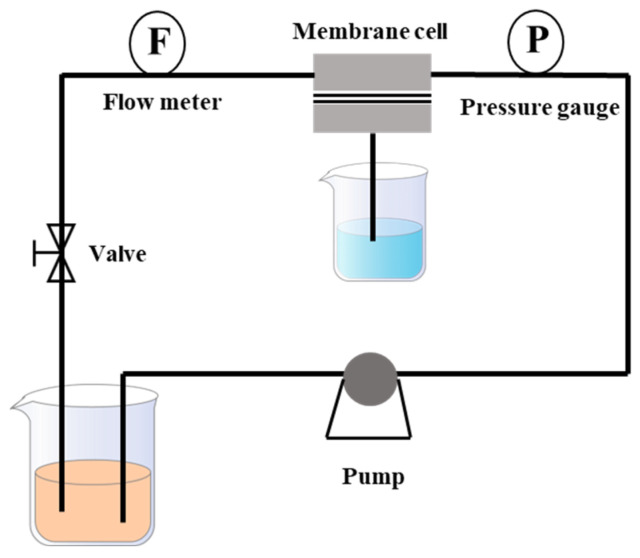
Schematic diagram of filtration device.

The filtration performance of membranes was texted using a home-made cross-flow instrument (Figure 2). Firstly, the prepared membranes were placed into the cross-flow device and pre-pressed with deionized water for 30 min under a pressure of 2.0 bar. Then, the membranes were tested at 1.0 bar with deionized water, with 100.0 ppm of dyes solution or 1000.0 ppm of salts solution as the feed. Each sample was tested at least three times, and the average value was taken as the final result.

The permeate flux (J, L m^−2^ h^−1^) of the membranes was calculated using Equation (1) [29].

J = V/At(1)

where V (L) is the volume of filtered water, t (h) is the time and A (m^2^) is the effective filtration area.

The rejection rate of the membranes was calculated using Equation (2) [29].

R (%) = 100 (C_f_ − C_P_)/C_f_ (2)

where C_p_ (g L^−1^) and C_f_ (g L^−1^) are the concentration of filtered and feed solutions, respectively. Dye concentrations were measured using an UV-vis spectrophotometer (UV 7, Mettler Toledo, Columbus, OH, USA) and a total organic carbon analyzer (TOC-L, Shimadzu, Kyoto, Japan). Salt concentrations were determined using a conductivity meter (FE 38, Mettler Toledo, Columbus, OH, USA).

### 2.6. Water Uptake Analysis of Membranes

The membranes were prepared with more MXene loading (10 mg); thus, for the GA-CM20 membrane, the CNC content is 2 mg. The PES membranes were not treated with PDA solution, so the MXene lamellar membranes could be peeled off from the substrates. The MXene lamellar membranes were soaking in 50 mL water for 2 h. The water uptake (W) of the membrane was calculated using Equation (3) [35].

W (%)= 100(m_1_ − m_0_)/m_0_ (3)

where m_0_ (g) and m_1_ (g) are the mass of dry membrane and wetted membrane, respectively.

### 2.7. Calculations of Activation Energy (Ea) of Membranes

The activation energy for water conduction through the MXene and GA-CM20 membranes was calculated using Equation (4) [36].

J_i_ = J_0_ e^−Ea/RT^ (4)

where J_i_ is the water permeate flux (L m^−2^ h^−1^ bar^−1^), J_0_ is the pre-exponential factor (L m^−2^ h^−1^ bar^−1^), E_a_ is the activation energy (kJ mol^−1^), R is the gas constant (R = 0.083145 kJ mol^−1^ K^−1^) and T is the temperature (K).

The logarithm was taken of both sides of Equation (5) [36].

ln(J_i_) = ln(J_0_) − (E_a_/8.3145)(1000/T) (5)

### 2.8. Membrane Stability Performance

The stability of the membranes was calculated by conducting cycle filtration measurements. Every cycle was tested for 3 h, then stored in water before the next measurement. The stability of the membranes was also conducted under different pressures (1–6 bar).

### 2.9. Membrane Anti-Fouling Performance

Bovine serum albumin (BSA) solution (1000.0 ppm) was chosen as the modern pollutant to study the anti-fouling property of the membrane. Firstly, the initial flux tested with DI water at 1.0 bar was named J_1_ (L m^−2^ h^−1^ bar^−1^). Secondly, pollutants solution was used as the feed and the obtained flux was named J_2_ (L m^−2^ h^−1^ bar^−1^). Lastly, the membrane was cleaned with DI water followed by DI water flux measurement, named J_3_ (L m^−2^ h^−1^ bar^−1^). Flux recovery (FRR) was calculated using Equation (6) to evaluate the anti-fouling performance of the prepared membranes.

FRR = J_3_/J_1_ (6)

The BSA adsorption testing was conducted with the method as reported in [35]. The membranes were placed into a filtration cell with 50 mL BSA solution (1.0 g/L). The static and dynamic adsorption experiments were conducted under non-stirring and stirring (200 rpm) conditions, respectively. After 12 h treatment, the concentrations of BSA in the solution before and after adsorption were measured with a UV-vis spectrophotometer (UV 7, Mettler Toledo, Columbus, OH, USA) at 278 nm and the amount of BSA adsorption (m, μg cm^−2^) was calculated using Equation (7).

m= V_a_ (C_0_ − C_1_)/A_a_ (7)

where V_s_ (mL) is 50 mL in this work, C_0_ and C_1_ (μg mL^−1^) were the BSA concentration at 0 h and 12 h, respectively, and A_a_ (cm^2^) is the membrane area. The reported data were the mean values of triplicate samples for each membrane.

## 3. Results

### 3.1. Characterization of Nanomaterial

**Figure 3:**
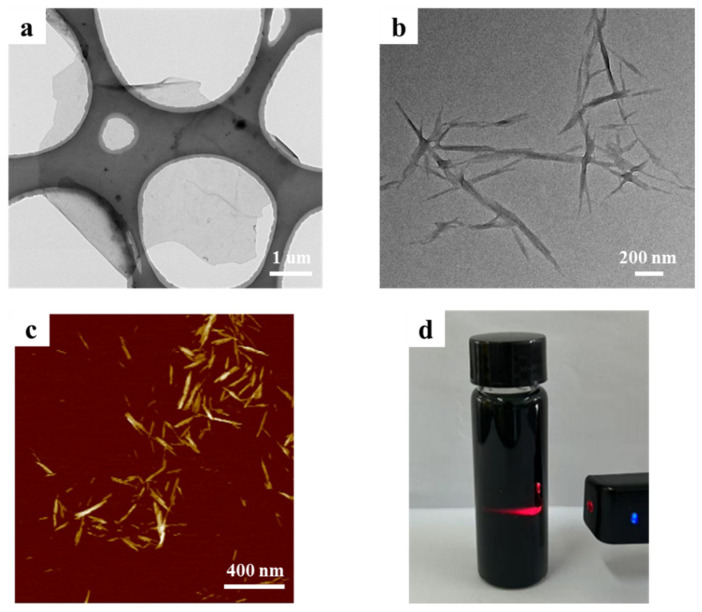
(**a**) TEM image of MXene nanosheet. (**b**) TEM image of CNC. (**c**) AFM image of CNC. (**d**) Tyndall effect of MXene/CNC mixture.

Ti_3_AlC_2_ powder was used as the precursor to prepare Ti_3_C_2_T_x_ MXene nanosheets using the etching method with a HCl/LiF mixture. Single-layer MXene nanosheets with lateral dimensions of ∼3.0 μm can be obtained after ultrasonic treatment (Figure 3a). Figure 3b,c show TEM and AFM images of the cellulose nanocrystal (CNC) with a diameter of ∼10.0 nm and a length of ∼400.0 nm. After mixing nanosheets and CNC in proportion, a uniform solution can be formed without precipitation. Meanwhile, the mixture exhibits a significant Tyndall effect (Figure 3d).

### 3.2. Characterization of Membranes

**Figure 4:**
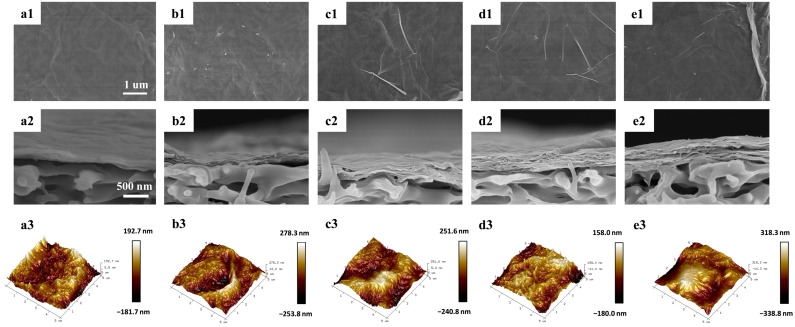
Surface SEM (magnification: 20,000), cross-sectional SEM (magnification: 40,000) and AFM images of (**a1**–**a3**) MXene membrane; (**b1**–**b3**) GA-CM10 membrane; (**c1**–**c3**) GA-CM20 membrane; (**d1**–**d3**) GA-CM30 membrane and (**e1**–**e3**) GA-CM40 membrane.

The MXene–CNC membranes were prepared by depositing mixture on porous PES membranes using the vacuum filtration method (Figure 1). After depositing the MXene nanosheets, no visible pores appeared on the membrane surface (Figure 4(a1)). The MXene membrane showed a typical wrinkled structure, which is caused by the stacking and shrinkage of nanosheets during the drying process. The MXene–CNC membranes exhibited a distinct roughness in surface structure (Figure 4(b1–e1)), which may be caused by the intercalation adjustment of CNC between MXene nanosheets. In addition, the needle-like structure on the surface of the film increases with the increased content of CNC. The interaction force between CNC and nanosheets may cause the aggregation of nanosheets and CNC, resulting in the irregular stacking of the membrane. The cross-sectional SEM images of prepared membranes are shown in Figure 4(a2–e2). The MXene–CNC composite membranes show looser stacking and a higher thickness than that of the pristine MXene membrane. The presence of CNC traversing the interlayer spaces of nanosheets contributes to the bulging of wrinkle folds and the creation of arched channels, resulting in loose and heterogeneous structures. Atomic Force Microscopy (AFM) images were used to measure the topological structure of the membrane surface (Figure 4(a3–e3)). The average surface roughness of the MXene, GA-CM10, GA-CM20, GA-CM30 and GA-CM40 is 42.5 nm, 53.6 nm, 61.4 nm, 66.7 nm and 71.1 nm, respectively, which is consistent with the results from the SEM images. Higher roughness can increase the filtration area of the membrane, but it can also decrease the anti-fouling performance of the membrane [37].

**Figure 5:**
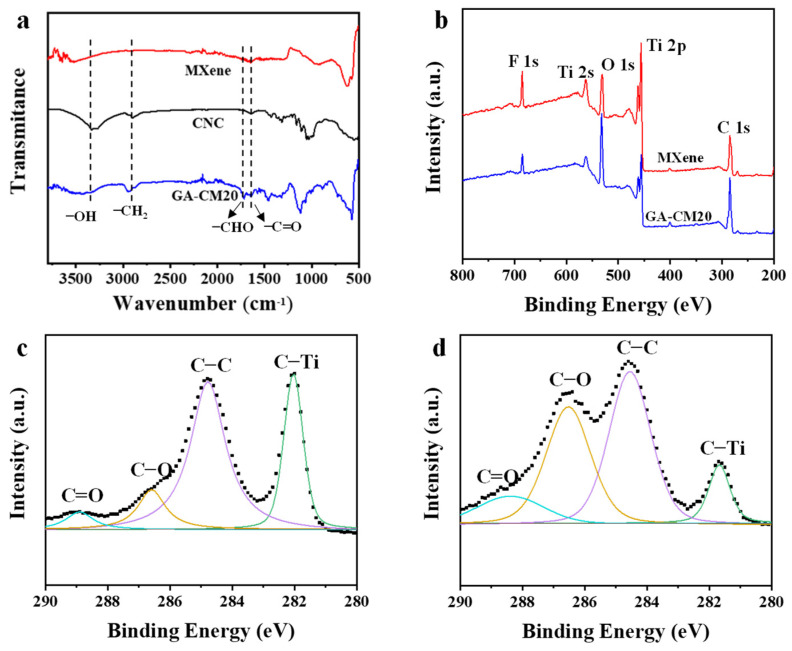
(**a**) FT-IR spectra of the MXene, CNC and GA-CM20 membranes. (**b**) XPS spectra of the MXene and GA-CM20 membranes. (**c**) C 1s core-level spectrum of the MXene membrane. (**d**) C 1s core-level spectrum of the GA-CM20 membrane.

To better understand the interactions within the composite membrane, functional groups were analyzed using FT-IR (Figure 5a). In the spectrum of MXene, the peaks at 3550 and 1630 cm^−1^ correspond to the stretching vibrations of the -OH and C=O bonds, respectively. For the composite membrane, the characteristic band at 2970 cm^−1^ is assigned to the C–H stretching vibration of the CNC and GA. Meanwhile, a new peak around 1730 cm^−1^ is attributed to the dangling aldehyde groups present in the membrane as a result of partial reactions. In addition, compared with the pristine MXene membrane, the -OH peak for the composite membrane is shifted to lower wavenumbers. This is attributed to the hydrogen bond formation between nanosheets and CNC, which will enhance the stability of the resulting membrane. Figure 5b shows the XPS survey spectrum of the MXene and GA-CM20 membranes. All the membranes show the C, O, F and Ti peak. After intercalation and cross-linking treatment, the O percentage is obviously increasing, while the Ti and F percentages are decreasing, which is attributed to the high O content of CNC molecules. Further information on chemical compositions was analyzed through high-resolution C 1s spectra. The high-resolution XPS spectrum of the C 1s of the MXene membrane shows four peaks at 281.9, 284.8, 286.7 and 288.5 eV, corresponding to C-Ti, C-C, C-O and C=O bonds, respectively (Figure 5c). For the GA-CM20 membrane (Figure 5d), the peak intensity of C-Ti decreases, while C-O increases. These changes in elements and chemical structure suggest the successful intercalation of CNC and cross-linking of GA.

**Figure 6:**
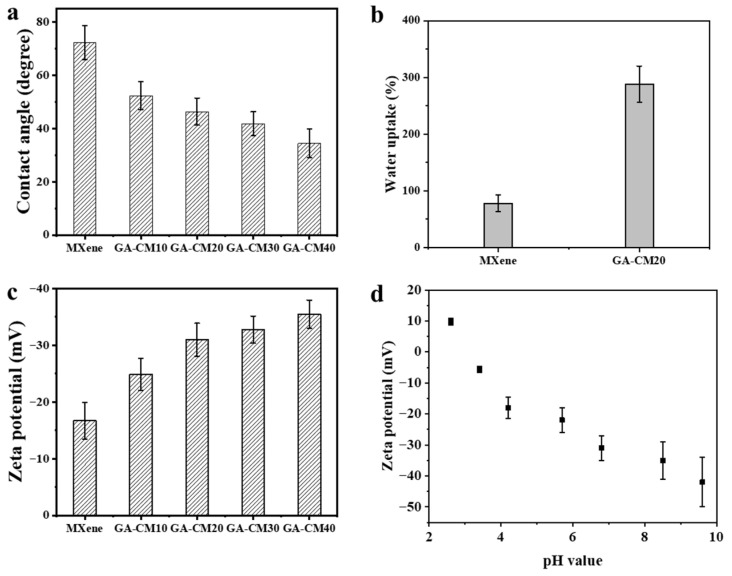
(**a**) Water contact angle of the prepared membranes. (**b**) Water uptake of the MXene and GA-CM20 membranes. (**c**) Surface zeta potential of the prepared membranes. (**d**) Surface zeta potential of the GA-CM20 membrane as a function of pH value.

The surface hydrophilicity of the membranes was evaluated using the static water contact angle. As displayed in Figure 6a, the water contact angle for the GA-CMx membrane is smaller than that of the pristine MXene membrane, which is attributed to the loading of CNC. In addition, the loose stacking structure also facilitates the rapid permeation of water molecules. The surface zeta potential measurement was studied to investigate the membrane surface charge properties. The water uptake results of the membranes are shown in Figure 6b; the GA-CM20 membrane has a higher water adsorption than that of the MXene membrane due to the CNC, which can fix more water molecules in the interlayer. As shown in Figure 6c, the pristine MXene membrane is electronegative at pH = 6.5, which is attributed to the existence of -OH, C=O and -F groups. After CNC loading and cross-linking with GA, a more negative surface potential for the composite membrane was detected. The electronegativity of the membrane surface increases with the increase in CNC concentration. In addition, Figure 6d shows that the GA-CM20 membrane is pH-sensitive and can be strengthened upon raising the pH value.

**Figure 7:**
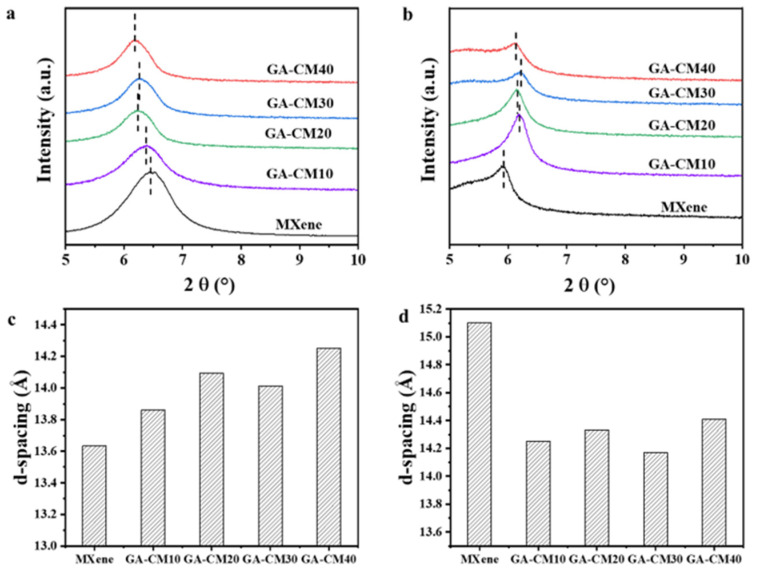
(**a**) XRD results of the dried membranes. (**b**) XRD results of the wetted membranes. (**c**) d-spacing of the dried membranes. (**d**) d-spacing of the wetted membranes.

XRD measurements were used to investigate the variation in interlayer spacing of the prepared membranes. The d-spacings values were calculated using Bragg’s law λ = 2dsinθ (λ = 0.154 nm). As shown in Figure 7a, compared to the pristine MXene membrane, the diffraction peaks of the composite membrane shift to a lower angle in the dry state, suggesting that the d-spacings increase from 13.6 to 14.3 Å (Figure 7c). The increased d-spacing observed in the composite membrane compared to the MXene membrane suggest that the addition of CNC enlarged the interlayer spaces between the MXene nanosheets. The wide channels enable water molecules to quickly pass through the 2D membrane. In the wet state, the d-spacings of the MXene membrane is 15.1 Å (Figure 7b,d), which is much higher than that of the composite membrane. Therefore, the intercalation and cross-linking treatment can effectively improve the anti-swelling performance of the membranes, thereby enhancing membrane stability. CNC is a good water absorbent and can fix more water molecules in the interlayer, resulting in full volume expansion. This expansion enlarges the interlayer spacing of its adjacent MXene nanosheets and prevents the MXene laminates from being packed too densely. At the same time, the GA cross-linker acts as a lock through covalent bonds to prevent the dissociation of adjacent nanosheets in water, which can explain the relatively steady d-spacing [38].

### 3.3. Separation Performance of Membranes

**Figure 8:**
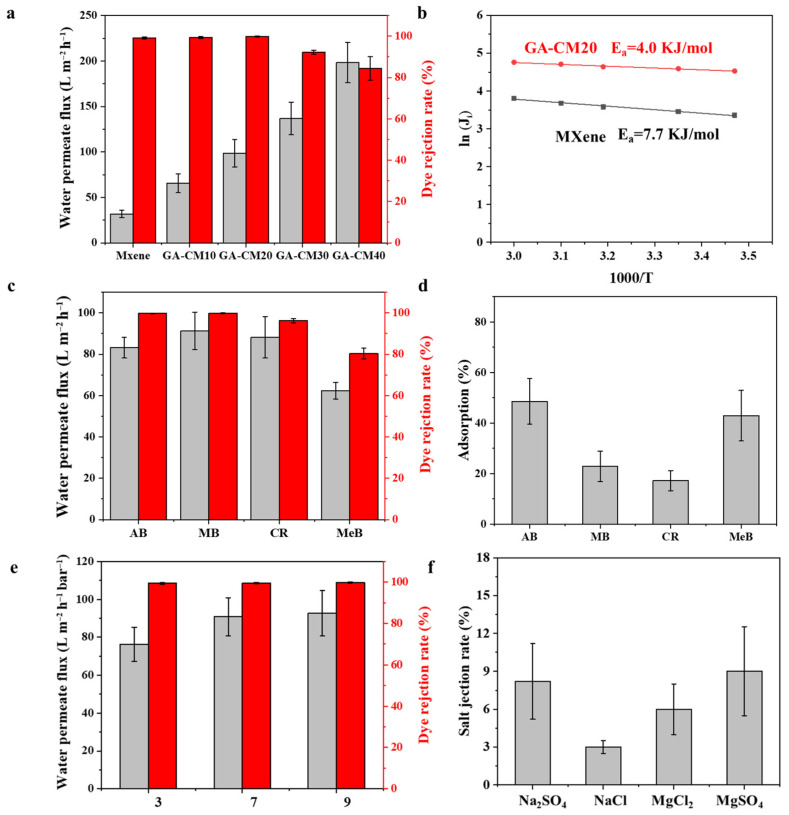
(**a**) Pure water permeate flux and MB rejection rate of prepared membranes (100.0 ppm of MB solution). (**b**) Activation energy of MXene and GA-CM20 membranes calculated using Arrhenius-type equation. (**c**) Permeate flux and rejection rate of GA-CM20 membrane using 100.0 ppm of different dyes solution as feed. (**d**) Dyes adsorption of GA-CM20 membrane (50.0 mL of 100.0 ppm of dye). (**e**) Effect of pH value of MB solution on GA-CM20 membrane performance. (**f**) Rejection rate of GA-CM20 membrane for different salts (1000.0 ppm).

The separation performance of the prepared membranes was evaluated in a home-made cross-flow device. As shown in Figure 8a, the pure water permeate flux of MXene-based membranes with different contents of CNC is distinctly enhanced, increasing from 31.8 to 198.2 L m^−2^ h^−1^. The improved permeate flux of the composite membrane could be attributed to the enhanced membrane surface roughness, surface hydrophilicity and interlayer spacing. The transmembrane energy barrier was used to measure the energy needed for water to pass through the membrane. As shown in Figure 8b, the activation energy value (E_a_) of the MXene/CNC composite membrane is lower than that of the pristine MXene membrane, suggesting that the composite membrane has a lower resistance to transmembrane water transport. Although the intercalation of CNC increases the size of the nanochannels, the cross-linking treatment inhibits the swelling of the nanosheets and reduces defects, thereby maintaining the high dye rejection rate. However, when the loading content of CNC is high (>30%), the MB rejection rate of the membrane decreases (Figure 8a). This may be attributed to the formation of arched channels and the gradual expansion of d-spacing in composite membranes, resulting in a decrease in the dye rejection rate. In addition, the aggregation of nanomaterials may lead to the formation of defects in the membrane, thus resulting in the poor selectivity. The modified membrane prepared under optimized conditions can simultaneously enhance water permeate flux and dye retention, thus overcoming the trade-off limitation. Among them, the GA-CM20 membrane was chosen for further separation experiments.

**Figure 9:**
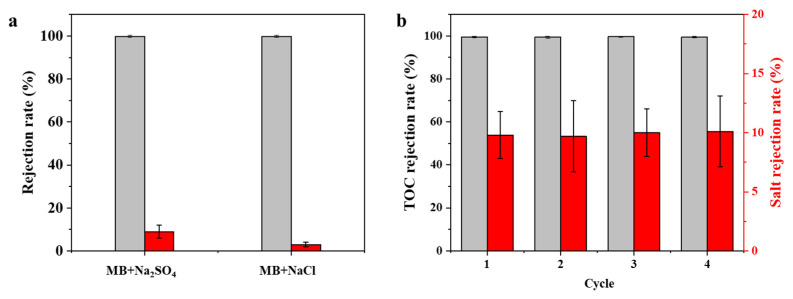
(**a**) Rejection rate of GA-CM20 membrane for mixture of dye and salt (100.0 ppm of dye and 100.0 ppm of salt). (**b**) Rejection rate of GA-CM20 membrane for MB/CR/Na_2_SO_4_ mixture (100.0 ppm of MB, 100.0 ppm of CR and 100.0 ppm of Na_2_SO_4_).

Four types of dyes with different molecular weight and charge properties were used to test the permselective performance of the GA-CM20 membrane. As shown in Figure 8c, the rejection rates of the GA-CM20 membrane for AB (M_w_ = 1298.9), MB (M_w_ = 799.8), CR (M_w_ = 696.7) and MeB (Mw = 319.1) are 98.3.0%, 99.6%, 97.0 and 80.2, respectively. This indicates that steric hindrance plays an important role during filtration. It was found that although positively charged AB has a higher molecular weight than negatively charged MB, the MB rejection rate is higher than that of AB. This also indicates that electrostatic repulsion contributes to a high dye rejection rate. In addition, the adsorption experiment shows that 48.6% of AB, 22.9% of MB, 17.2% of CR and 42.9% of MeB are adsorbed by the membrane within 6 h (Figure 8d), suggesting that adsorption also plays an important role in the rejection rate, especially of cationic dyes. The effect of the pH value of the solution on membrane performance was also studied. As shown in Figure 8e, with increasing pH values, the MB rejection rate has almost no change. Under low pH conditions, the polarity and solubility of MB (pKa = 5) are reduced due to the protonation of sulfonated acid groups. Although the electronegative membrane will adsorb dye molecules through electrostatic forces, the aggregation of dye molecules enhances the size sieving effect, thus maintaining a high dye rejection rate. The adsorbed dye blocks the membrane pores, resulting in a decrease in membrane flux. To summarize, the separation mechanism is expected to be complex, likely caused by the synergetic effects of the electrostatic repulsion effect, size sieving, adsorption and molecule aggregation of the dyes. As shown in Figure 8f, the GA-CM20 membrane shows a lower salt rejection rate (<10%). Salt/dye and dye/dye mixture solutions were also used to explore the separation performance. As shown in Figure 9a,b, the GA-CM20 membrane shows a high dye rejection rate and low salt rejection rate for the mixture solutions. Under this premise, its superior permeate flux would make it suitable for textile wastewater treatment. More importantly, compared with the state-of-the-art MXene-based membranes (Table 2), the GA-CM20 membrane features a good combination of high permeate flux, selectivity and anti-fouling properties.

### 3.4. Long-Term Stability and Anti-Fouling Performance of Membranes

**Figure 10:**
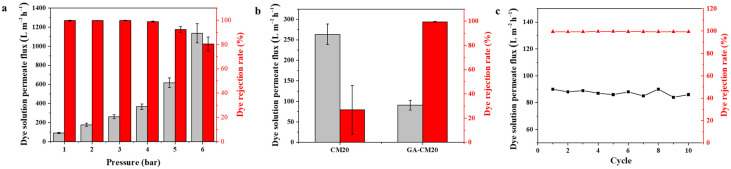
(**a**) Permeate flux and rejection rate of GA-CM20 membrane under various operating pressures. (**b**) Stability performance of CM20 and GA-CM20 membrane under 3 bar pressure. (**c**) Long-term stability of GA-CM20 membrane.

As shown in Figure 10a, the permeate flux and MB rejection rate of GA-CM20 membrane is stable when the pressure is less than 4 bar. However, when the pressure exceeds 4 bar, the permeate flux begins to increase and rejection rate begins to decrease. This is because the shedding of nanosheets under high pressure causes defects in the membrane, which increases the water permeate flux and decreases the MB rejection rate. Therefore, this membrane is suitable for use at low pressure. Meanwhile, the composite membrane without cross-linking treatment was also used to explore the stability performance. As displayed in Figure 10b, there is a significant difference in separation performance between the CM20 and GA-CM20 membranes. The intercalation of CNC can expand the interlayer spacing between adjacent MXene nanosheets for water transportation. However, the weak interaction between CNC and MXene cannot prevent the nanosheets from dissociating in water, especially under cross-flow mode. Thus, cross-linking treatment is an important step for providing the membranes with high stability. In addition, cycle filtration testing for the GA-CM20 membrane confirms its long-term stability (Figure 10c).

**Figure 11:**
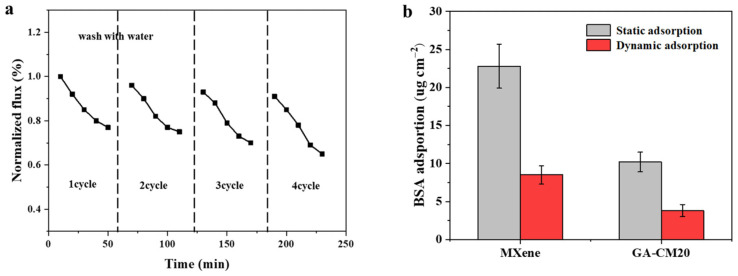
(**a**) Anti-fouling performance of GA-CM20 membrane with BSA as the model pollutant (1.0 g/L). (**b**) BSA adsorption measurement of MXene and GA-CM20 membranes.

The anti-fouling performance of membranes is crucial for their practical application. As shown in Figure 11a, during the first filtration cycle for treating BSA solution, the water permeate flux of the GA-CM20 membrane decreases by 20%. BSA can deposit on the membrane’s surface, leading to a decrease in permeate flux. After washing with the DI water, the flux recovery ratio (FRR) of GA-CM20 membrane is about 96% and over 90% after a four-cycle filtration experiment, indicating that the GA-CM20 membrane has good anti-fouling properties. As shown in Figure 11b, compared with the MXene membrane, a lower amount of BSA adheres to the GA-CM20 membrane surface under both dynamic and static conditions, also accounting for its good anti-fouling performance. Generally, the anti-fouling performance of a membrane is greatly related to the membrane’s surface properties, such as surface hydrophilicity, roughness and charge. The hydrophilic CNC can adsorb water molecules to form a hydration layer to resist the adhesion of foulants. In addition, the electrostatic repulsion effect between negatively charged foulants and the membrane surface also contribute to the high anti-fouling performance.

## 4. Conclusions

In conclusion, a range of GA-CMx composite membranes were prepared using intercalation and a cross-linking reaction. The intercalation of CNC enlarges the nanochannels of the MXene membrane; the subsequent cross-linking treatment further inhibits membrane swelling and defect formation, endowing the membrane with the excellent combination of characteristics of high permeate flux and selectivity. The water permeate flux of the optimized GA-CM20 membrane is 3.1 times improved, which is attributed to the expanded nanochannels and enhanced hydrophilicity. Meanwhile, the reduced defects and regulable nanochannels provide the membrane with a high dye rejection rate (>97%) and a low salt rejection rate (<10%). Furthermore, the GA-CM20 membrane exhibits excellent stability and anti-fouling performance. This work presents an efficient approach for developing a high-performance 2D membrane, which might be of interest in many promising applications in energy- and environment-related fields.

## Figures and Tables

**Table 1:** Composition of prepared membranes.

Membrane	MXene Loading (mg)	CNC Loading (mg)	CNC/MXene Ratio (%)
MXene	1.0	0	0
GA-CM10	1.0	0.1	10
GA-CM20	1.0	0.2	20
GA-CM30	1.0	0.3	30
GA-CM40	1.0	0.4	40

**Table 2:** Performance comparison of the related membranes.

Membrane	Water Permeate Flux (LMH/bar)	Dye Rejection Rate	FRR	Reference
GA-CM20	90.8	MB: 99.6% CR: 97.2%	BSA 96%	This work
MCE/MXene	44.97	MeB: 100.0%	N *	[39]
MXene/PEI/TMC	38.2	CR: 99.7%	N *	[40]
MXene/enzymatic hydrolysis lignin	61.22	MB:78.5%	Methylene blue 90.33%	[17]
MXene/PEI	20.9	CR: 99.42%	N *	[18]
MXene/phytic acid	510	CR:99.7%	N *	[41]
GO/MXene-PPS	52.8	MB:99.9%	Methyl blue 85%	[42]

* Not mentioned in the paper.

## Data Availability

The original contributions presented in this study are included in the article. Further inquiries can be directed to the corresponding author.

## Abbreviations

The following abbreviations are used in this manuscript:

MXene	carbides and nitrides
Ti_3_C_2_T_x_	Titanium carbide
CNC	Cellulose nanocrystal
2D	Two-dimensional
GO	Graphene oxide
MOF	Metal–organic framework
COF	Covalent organic framework
PDA	Polydopamine
GA	Glutaraldehyde
PES	Polyethersulfone
BSA	Bovine serum albumin
AB	Alcian blue
MB	Methyl blue
CR	Congo red
MeB	Methylene blue
Tris-HCl	Tris (hydroxymethyl) aminomethane hydrochloride

## References

[1] Ma H., Yu L., Yang L., Yao Y., Shen G., Wang Y., Li B., Meng J., Miao M., Zhi C. (2025). Graphene oxide composites for dye removal in textile, printing and dyeing wastewaters: A review. Environ. Chem. Lett..

[2] Samuchiwal S., Gola D., Malik A. (2021). Decolourization of textile effluent using native microbial consortium enriched from textile industry effluent. J. Hazard. Mater..

[3] Tang H., Shan M., Cheng Q., Wen R., Liu S., Zeng H., Yu J., Luo J. (2024). Rapid pretreatment strategy to control nanofiltration membrane fouling in recycling of real textile wastewater: Comparison and mechanisms. Chem. Eng. J..

[4] Liu Y., Zhu J., Chi M., Eygen G.V., Guan K., Matsuyama H. (2025). Comprehensive review of nanofiltration membranes for efficient resource recovery from textile wastewater. Chem. Eng. J..

[5] Lin S., Qi H., Hou P., Liu K. (2023). Resource recovery from textile wastewater: Dye, salt, and water regeneration using solar-driven interfacial evaporation. J. Clean. Prod..

[6] Zhai M., Peng H., Li K. (2024). High-performance loose nanofiltration membranes with excellent antifouling properties for dye/salt separation. J. Membr. Sci..

[7] Tang Y., Yu H., Zhang Y., Liu J., Zhu J., Lin Y., Chen H., Hu Y., Zhang J., Hu S. (2025). Preparation of a polysulfone loose nanofiltration membrane with gradient structure via hybrid-induced phase separation for the efficient separation of dyes/salts. Desalination.

[8] Sun L., Jia H., Gao F., Wang J. (2025). Insights into the performance gap in nanofiltration-based dye separation: Laboratory vs. factory perspectives. Chem. Eng. Sci..

[9] Dmitrenko M., Sushkova X., Chepeleva A., Liamin V., Mikhailovskaya O., Kuzminova A., Semenov K., Ermakov S., Penkova A. (2023). Modification approaches of polyphenylene oxide membranes to enhance nanofiltration performance. Membranes.

[10] Zheng J., Liu Y., Zhu J., Jin P., Croes T., Volodine A., Yuan S., Van der Bruggen B. (2021). Sugar-based membranes for nanofiltration. J. Membr. Sci..

[11] Lv Z., Li H., Wen C., Tian L., Chen X., Wu W., Li Z. (2025). 2D biomimetic membranes constructed by charge assembly and hydrogen bonding for precise ion separation. Adv. Mater..

[12] Karahan H.E., Goh K., Zhang C., Yang E., Yıldırım C., Chuah C.Y., Ahunbay M.G., Lee J., Tantekin-Ersolmaz Ş.B., Chen Y. (2020). MXene materials for designing advanced separation membranes. Adv. Mater..

[13] Xu Y., Zhao X., Chang R., Qu H., Xu J., Ma J. (2022). Designing heterogeneous MOF-on-MOF membrane with hierarchical pores for effective water treatment. J. Membr. Sci..

[14] Sapkota B., Liang W., VahidMohammadi A., Karnik R., Noy A., Wanunu M. (2020). High permeability sub-nanometre sieve composite MoS2 membranes. Nat. Commun..

[15] Zhang X., Zhao X., Sun J., He Y., Wu B., Ge L., Pan J. (2025). Ultrathin zwitterionic COF membranes from colloidal 2D-COF towards precise molecular sieving. Water Res..

[16] Khosla A., Sonu, Awan H.T.A., Singh K., Gaurav, Walvekar R., Zhao Z., Kaushik A., Khalid M., Chaudhary V. (2022). Emergence of MXene and MXene–polymer hybrid membranes as future- environmental remediation strategies. Adv. Sci..

[17] Zhang X., Cheng S., Li R., Guo Z., Zhao Y., Sun Y., Chen Y., Nie L., Li Z. (2025). Hybrid lignin-intercalated MXene membranes for enhanced water purification. Sep. Purif. Technol..

[18] Li J., Li L., Xu Y., Zhu J., Liu F., Shen J., Wang Z., Lin J. (2022). MXene nanosheet stacks with tunable nanochannels for efficient molecular separation. Chem. Eng. J..

[19] Xie L., Tang J., Qin R., Zhang Q., Liu J., Jin Y., Wang H. (2023). Surface charge modification on 2d nanofluidic membrane for regulating ion transport. Adv. Funct. Mater..

[20] Su Z., Malankowska M., Marschall Thostrup T., DeMartini M., Khajavi P., Guo H., Storm Pedersen L., Pinelo M. (2024). Comparison of 2D and 3D materials on membrane modification for improved pressure retarded osmosis (PRO) process. Chem. Eng. Sci..

[21] Zou J., Wu J., Wang Y., Deng F., Jiang J., Zhang Y., Liu S., Li N., Zhang H., Yu J. (2022). Additive-mediated intercalation and surface modification of MXenes. Chem. Soc. Rev..

[22] Wang J., Zhang Z., Zhu J., Tian M., Zheng S., Wang F., Wang X., Wang L. (2020). Ion sieving by a two-dimensional Ti_3_C_2_T_x_ alginate lamellar membrane with stable interlayer spacing. Nat. Commun..

[23] Zhang Z., Yang S., Zhang P., Zhang J., Chen G., Feng X. (2019). Mechanically strong MXene/Kevlar nanofiber composite membranes as high-performance nanofluidic osmotic power generators. Nat. Commun..

[24] Xu R., Kang Y., Zhang W., Pan B., Zhang X. (2023). Two-dimensional MXene membranes with biomimetic sub-nanochannels for enhanced cation sieving. Nat. Commun..

[25] Long Q., Zhao S., Chen J., Zhang Z., Qi G., Liu Z.-Q. (2021). Self-assembly enabled nano-intercalation for stable high-performance MXene membranes. J. Membr. Sci..

[26] Luo H., Xu N., Li Y., Li J., Ji W., Nian P., Wang Z., Wei Y. (2024). Amino acid-bonded Ti_3_C_2_Tx MXene nanofiltration membranes with superior antifouling property for enhanced water purification. J. Membr. Sci..

[27] Zheng Y., Zhang H., Yu S., Zhou H., Chen W., Yang J. (2024). Covalently bridged MXene/COF hybrid membrane toward efficient dye separation. Sep. Purif. Technol..

[28] Zeng Q., Zhao D.L., Shen L., Lin H., Kong N., Han L., Chen C., Teng J., Tang C., Chung T.-S. (2023). Titanium oxide nanotubes intercalated two-dimensional MXene composite membrane with exceptional antifouling and self-cleaning properties for oil/water separation. Chem. Eng. J..

[29] Qiu M., Shen Z., Xia Q., Li X., Huang H., Wang Y., Liu Y., Wang Y. (2022). Metal-polyphenol cross-linked titanium carbide membranes with stable interlayer spacing for efficient wastewater treatment. J. Colloid Interface Sci..

[30] Sun W., Yao Y., Ju J., Yuan H., Tan Y. (2025). Green, Cross-Linked, Curcumin-loaded konjac glucomannan/cellulose nanocrystal nanofiber membranes. ACS Appl. Polym. Mater..

[31] Gao H., Wang Y., Afolabi M.A., Xiao D., Chen Y. (2021). Incorporation of cellulose nanocrystals into graphene oxide membranes for efficient antibiotic removal at high nutrient recovery. ACS Appl. Mater. Interfaces.

[32] Ozbey-Unal B., Balcik C., Yuan S., Van der Bruggen B. (2025). Fabrication of cellulose nanocrystals-incorporated dense Janus membranes for enhanced desalination and oily saline wastewater treatment via membrane distillation. J. Membr. Sci..

[33] Bai L., Ding A., Li G., Liang H. (2022). Application of cellulose nanocrystals in water treatment membranes: A review. Chemosphere.

[34] Zhang Y., Chen D., Li N., Xu Q., Li H., He J., Lu J. (2022). High-performance and stable two-dimensional MXene-polyethyleneimine composite lamellar membranes for molecular separation. ACS Appl. Mater. Interfaces.

[35] You X., Wu H., Zhang R., Su Y., Cao L., Yu Q., Yuan J., Xiao K., He M., Jiang Z. (2019). Metal-coordinated sub-10 nm membranes for water purification. Nat. Commun..

[36] Zhang M., Zhao P., Li P., Ji Y., Liu G., Jin W. (2021). Designing biomimic two-dimensional ionic transport channels for efficient ion sieving. ACS Nano.

[37] Fu W., Chen J., Li C., Jiang L., Qiu M., Li X., Wang Y., Cui L. (2021). Enhanced flux and fouling resistance forward osmosis membrane based on a hydrogel/MOF hybrid selective layer. J. Colloid Interface Sci..

[38] Zandi Z., Rastgar M., Mohseni M., Firouzjaei M.D., Dilokekunakul W., Anasori B., Vecitis C.D., Keller R., Wessling M., Elliott M. (2024). Electro-conductive Ti_3_C_2_ MXene multilayered membranes: Dye removal and antifouling performance. Adv. Funct. Mater..

[39] Zhang S., Liao S., Qi F., Liu R., Xiao T., Hu J., Li K., Wang R., Min Y. (2020). Direct deposition of two-dimensional MXene nanosheets on commercially available filter for fast and efficient dye removal. J. Hazard. Mater..

[40] Gu S., Ma Y., Zhang T., Yang Y., Xu Y., Li J. (2023). MXene nanosheet tailored bioinspired modification of a nanofiltration membrane for dye/salt separation. ACS EST Water.

[41] Yi M., Héraly F., Chang J., Khorsand Kheirabad A., Yuan J., Wang Y., Zhang M. (2021). A transport channel-regulated MXene membrane via organic phosphonic acids for efficient water permeation. Chem. Commun..

[42] Ma X.-Y., Fan T.-T., Wang G., Li Z.-H., Lin J.-H., Long Y.-Z. (2022). High performance GO/MXene/PPS composite filtration membrane for dye wastewater treatment under harsh environmental conditions. Compos. Commun..

